# A novel method for monitoring the constancy of beam path accuracy in CyberKnife†

**DOI:** 10.1002/acm2.12585

**Published:** 2019-04-19

**Authors:** Bin Yang, Wing Kei Rebecca Wong, Wai Wang Lam, Hui Geng, Chi Wah Kong, Kin Yin Cheung, Siu Ki Yu

**Affiliations:** ^1^ Medical Physics and Research Department Hong Kong Sanatorium & Hospital Hong Kong Hong Kong

**Keywords:** ArcCHECK, beam path accuracy, CyberKnife, quality assurance

## Abstract

The aim of current work was to present a novel evaluation procedure implemented for checking the constancy of beam path accuracy of a CyberKnife system based on ArcCHECK. A tailor‐made Styrofoam with four implanted fiducial markers was adopted to enable the fiducial tracking during beam deliveries. A simple two‐field plan and an isocentric plan were created for determining the density override of ArcCHECK in MultiPlan and the constancy of beam path accuracy respectively. Correlation curves for all diodes involved in the study were obtained by analyzing the dose distributions calculated by MultiPlan after introducing position shifts in anteroposterior, superoinferior, and left–right directions. The ability of detecting systematic position error was also evaluated by changing the position of alignment center intentionally. The one standard deviation (SD) result for reproducibility test showed the RMS of 0.054 mm and the maximum of 0.263 mm, which was comparable to the machine self‐test result. The mean of absolute value of position errors in the constancy test was measured to 0.091 mm with a SD of 0.035 mm, while the root‐mean‐square was 0.127 mm with a SD of 0.034 mm. All introduced systematic position errors range from 0.3 to 2 mm were detected successfully. Efficient method for evaluating the constancy of beam path accuracy of CyberKnife has been developed and proven to be sensitive enough for detecting a systematic drift of robotic manipulator. Once the workflow is streamlined, our proposed method will be an effective and easy quality assurance procedure for medical physicists.

## 1 INTRODUCTION

Stereotactic radiosurgery (SRS) or fractioned stereotactic body radiotherapy (SBRT) has gained widespread applications in treating tumors in brain, lung, liver, spine, and kidney with favorable treatment outcomes.^1^, ^2^, ^3^, ^4^, ^5^ As a complex procedure involving frame‐based or frameless immobilization, SRS/SBRT delivers small beams in multiple noncoplanar arcs to achieve a highly conformal dose distribution on the targets, while minimizing the dose to the surrounding normal tissue. Among many advanced techniques, the CyberKnife robotic radiosurgery system (Accuray Inc., Sunnyvale, CA, USA) has been increasingly employed for the SRS and SBRT.^6^, ^7^, ^8^ This treatment system consists of a 6 MV flattening‐filter free linear accelerator mounted on an industrial frameless robotic arm (Kuka, Augsburg, Germany). It is capable of delivering precise ablative radiation dose to the target by utilizing a large number of noncoplanar beams while simultaneously tracking target motion in real time.

After field service engineers (FSE) perform a full set of beam path calibration for all collimators, isocentric end‐to‐end (E2E) tests are performed to determine the overall targeting accuracy and coordinate coincidence of the CyberKnife system for each tracking method. In order to minimize the overall targeting error, a targeting correction value known as “DeltaMan” is introduced based on the E2E results to change the offset between the machine center of robot frame and the imaging center of target localization system (TLS) frame. Targeting accuracy is defined as the offset between centroid of the delivered 70% isodose line and the known centroid position in patient reference frame. The targeting error tolerance is 0.95 mm for all tracking methods. E2E tests are conducted for evaluating the performance of the overall system and the final results are influenced by both beam path accuracy and TLS. While the methodology for QA of TLS has been well established, simple and convenient QA methods for beam path accuracy still remain to be developed.

AAPM Task Group 135 recommends three levels of QA evaluations for the current state of manipulator‐pointing accuracy.^9^ The first level is either laser alignment check on the floor and/or automatic quality assurance (AQA) test, which is a simplified Winston‐Lutz test consisting of only two beams. The second level is to run a “BB test” (an isocentric plan) in simulation mode for visually checking whether the centerline laser fully illuminates the isocrystal tip. The third level is a quantitative evaluation of the second level test that is capable of recording node‐by‐node deviations. Both level 2 and level 3 tests are strongly based on the position of the beam central axis laser and level three can only be done with the assistance of a FSE. Because there is no alternative QA method for checking the pointing accuracy of individual node quantitatively, the Task Group recommended the development of a QA procedure that could be conducted easily and safely by a Qualified Medical Physicist.

A commercially available three‐dimensional (3D) cylindrical diode array ArcCHECK (SunNuclear Corp., Melbourne, FL) has been shown to be a useful tool for QA of Intensity Modulated Radiation Therapy (IMRT), Volumetric Modulated Arc therapy (VMAT) and Tomotherapy treatments.^10^, ^11^, ^12^, ^13^ The ArcCHECK consists of 1386 diode detectors which are arranged in a spiral pattern with length of 21 cm and diameter of 21 cm. In addition to its application in dosimetric verification, its unique 3D design also makes it a potential tool for machine QA.^14^, ^15^, ^16^ Although the ArcCHECK has been widely used for QA of isocentric treatment machines, its applications in commissioning of Monte Carlo algorithm and patient‐specific QA for nonisocentric treatment machine such as CyberKnife were also studied by some groups.^17^, ^18^ In this study, we developed a novel method for testing the constancy of beam path accuracy of our CyberKnife (model M6) system by using ArcCHECK. Though it is not intuitive for a detector array with a spatial resolution of 1 cm to detect a submillimeter position shift, we should emphasis that it is the single diode with high signal drawing our attention in this study. The position error was calculated based on the correlation curves and measured dose difference of a single diode instead of the conventional profile measured by multiple diodes.

## 2 MATERIALS AND METHODS

### 2.A Solution for fiducial tracking

**Figure 1:**
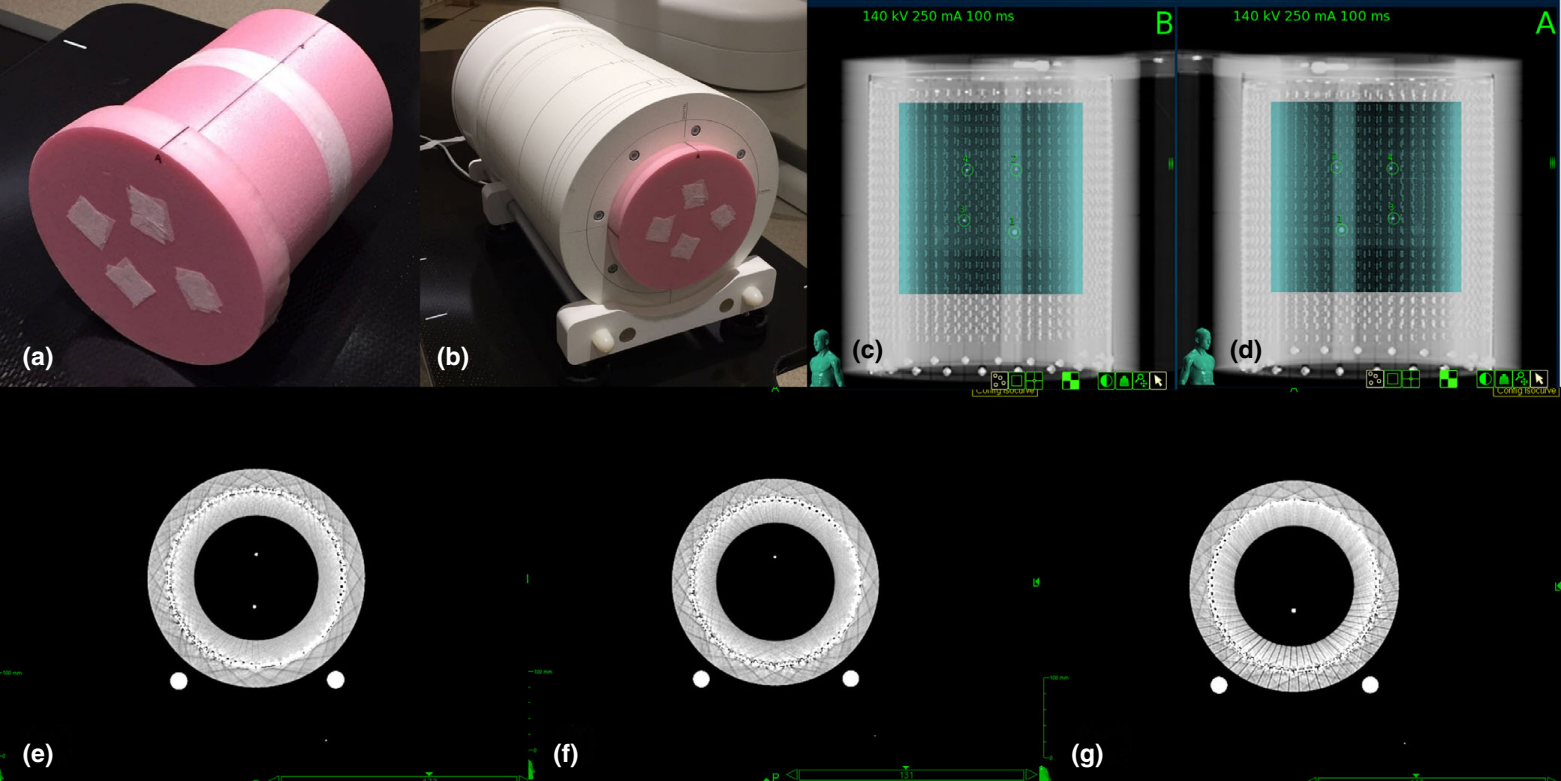
Photos of (a) tailor‐made Styrofoam with four implanted fiducial markers, (b) ArcCHECK device with the Styrofoam insert, (c), (d) Setup images of the ArcCHECK, (e)–(g) computed tomography images of the ArcCHECK with fiducials.

As shown in Figs. 1(a) and 1(b), a Styrofoam insert with four implanted computed tomography (CT) fiducial markers (Beekley Corporation, Bristol, CT, USA) was tailor‐made for the ArcCHECK. The whole device was scanned by a CT simulator (Siemens AG, Munich, Germany) using 120 kVp and 400 mAs. The four markers could be clearly located from the CT images, as indicated in Figs. 1(e)–1(g), which allowed the use of fiducial‐based tracking technique for all treatment plans created in this study. Figures 1(c) and 1(d) show the kV X‐ray images taken during treatment setup to demonstrate that the markers could be successfully distinguished from the high‐Z diodes of the ArcCHECK. Before each delivery of QA plan, the residual robot corrections for compensating the setup uncertainty were not larger than 0.1 mm and 0.1°, which reached the limitation of the precision of TLS.

### 2.B Density override and QA plans

To avoid the dosimetric uncertainty arising from the artifacts of kV CT images, the acrylic body of ArcCHECK was contoured and assigned a fixed relative electron density (RED). Since MultiPlan (CyberKnife treatment planning system) forced the RED values of air to be 0 for CT numbers between 0 and 199 inclusively,^19^ we also fixed the RED of the Styrofoam. A simple two‐field plan, which is similar to the conventional AQA plan, was created using only the 60 mm fixed cone. It consists of two manually selected orthogonal beams of 200 MU each [in anteroposterior (AP) and lateral direction with SSD = 65.2 and 81.5 cm respectively]. It was performed to find the most proper RED values which gave the minimum difference between the calculated and measured results.

**Figure 2:**
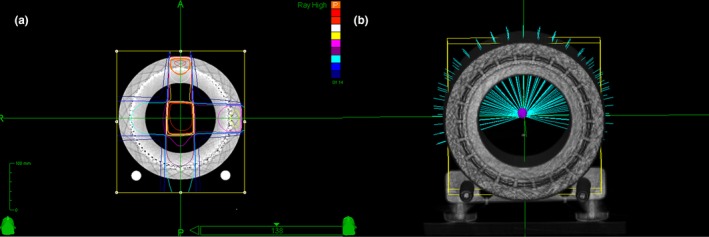
(a) Beam trajectories of the two‐field plan for verifying the designated relative electron density of PMMA and Styrofoam; (b) three‐dimensional views of the isocentric plan for monitoring the constancy of beam path accuracy.

To evaluate the constancy of beam path accuracy, an isocentric plan was created with a fixed 5 mm treatment cone. Total 116 beams in body path were selected, and 50 MU was assigned for each beam. Figure 2 shows the two QA plans generated by the Multiplan. In addition, a simple anterior beam with 200 MU was delivered every time before the delivery of QA plan to make sure the output constancy of CyberKnife was better than 0.5%. The accuracy of dose calibration of the ArcCHECK was verified by a routine check using a conventional LINAC with a 10 × 10 field size.

**Figure 3:**
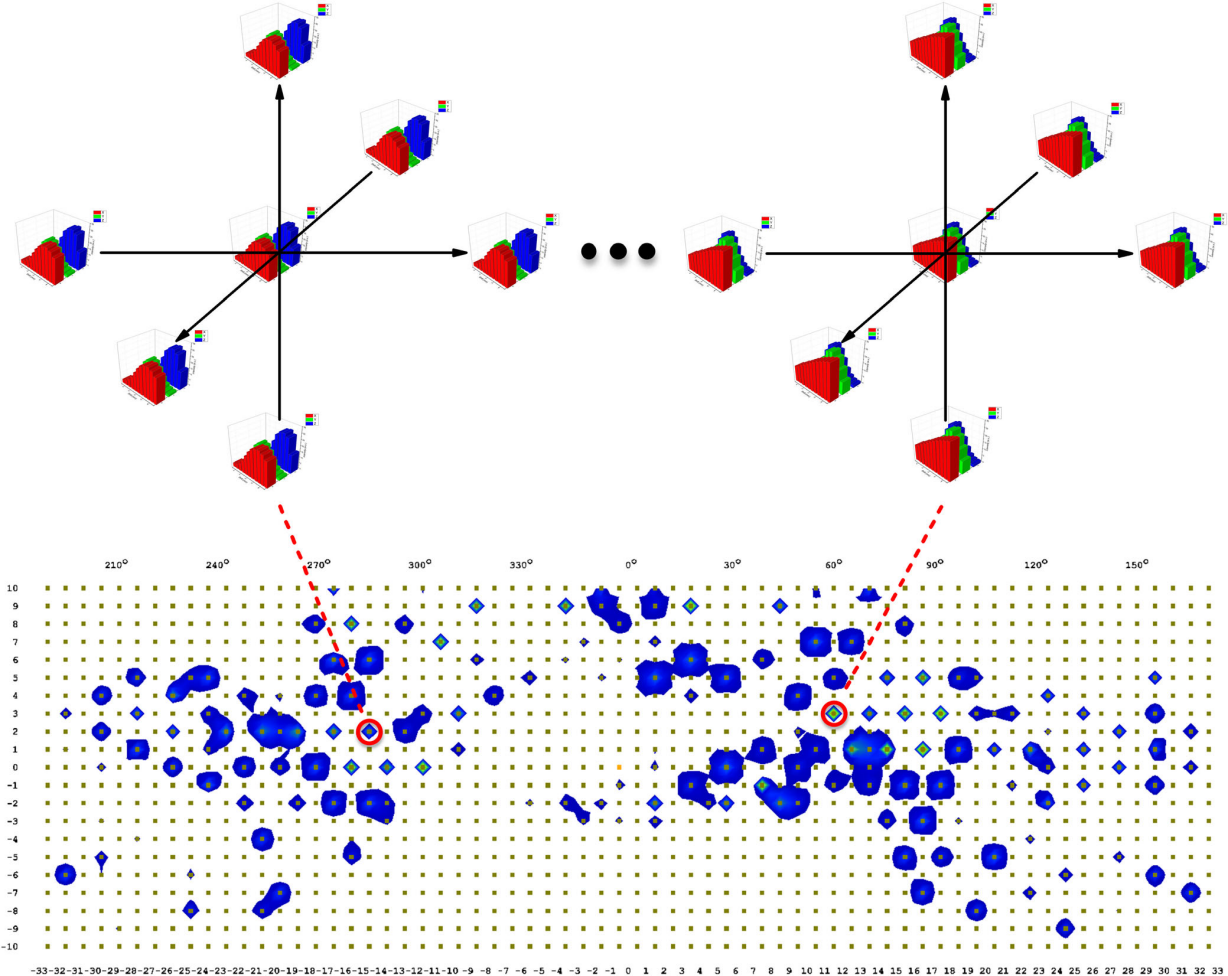
Illustration of the principle for creating the correlation curves for each diode. The red, green, and blue histograms plotted in 3D present the correlation curves in left–right, superioinferior, and anterioposterior direction respectively. The drawn coordinates stand for the different locations of diode when taking its location uncertainty into consideration.

### 2.C Correlation curves of diodes

To make sure the number of tracked beams coincided with the number of selected diodes, a complete delivery of the test plan using ArcCHECK was first saved as a movie file (.*acm* file) and the screen of console computer showing the beam ID was simultaneously recorded using a video camera. When analyzing the movie file, we set several criteria to make sure that each selected diode corresponded to one beam: (a) if multiple diodes succeeded to measure the signal from one beam, only the diode with the maximum signal would be selected; (b) if one diode picked up multiple signals from different beams, this diode would not be selected. Therefore, after the movie file was synchronized with the video, the connections between selected diodes and specific beams would be confirmed. In addition, to avoid those diodes too close to the field edge, a threshold of 10% was applied, which implied that only those diodes with measured signal larger than 10% of the maximum could be selected in the analysis. Finally a Look‐Up‐Table (LUT) containing a total of 68 selected diodes was saved and would be used for all future measurements.

The same CT images of ArcCHECK were anonymized and saved as a QA template in MultiPlan. Based on the isocentric plan mentioned above, we created a QA plan and then intentionally introduced position shifts that ranged from −5 to 5 mm with an interval of 0.25 mm between −1 and 1 mm in AP, superoinferior (SI) and left–right (LR) directions. Thus, a total of 49 DICOM dose files were generated and exported for analysis, which made it possible to create the correlation curves of dose variation vs position shift in three directions for selected diodes, as illustrated in Fig. 3.

Those DICOM dose files from MultiPlan were interpreted by the ArcCHECK software (SNC Patient), which is commonly used in IMRT/VMAT QA for comparing measured dose points to planned dose points. Planned dose in the location of any given diode could be easily extracted from each DICOM dose file and a simple MATLAB program was developed to create the correlation curves when given the location of a specific diode. According to the specifications of ArcCHECK,^20^ the placement accuracy of diode was 0.5 mm. Therefore, additional six sets of dose distributions were calculated by applying a shift of 0.5 mm to the DICOM center in all three directions when loading the DICOM dose file into the ArcCHECK software. A total of twenty‐one correlation curves were finally obtained for each selected diode.

As indicated above, the most time‐consuming part of our proposed method was the export and processing of DICOM dose files. Depending on the specifications of computers, 3–4 h may be needed to obtain all necessary files.

### 2.D Calculation of position error

The isocentric plan was delivered six times within 1 month and a baseline was then set by averaging these measurements. By comparing with the baseline, a map of relative percentage difference could be obtained for each measurement thereafter. Based on the dose difference map and correlation curves, position errors could be calculated in several steps while its rationality would be discussed later:

Step 1: For each set of correlation curves, three different position errors could be calculated in AP, SI, and LR directions. Because there was no way for us to figure out which direction the position error came from and some calculated errors in AP and LR directions were unrealistic due to relatively flat correlation curves, we considered the minimum position error in three directions as the ‘true’ position error. More elaborations could be found in Section 2.B.
Step 2: In order to reduce the influence from the placement inaccuracy of each diode, the isocentric plans was delivered five times consecutively on the same day. Based on the assumption that CyberKnife has good reproducibility for continuous deliveries without changing the setup, a map of standard deviation (SD) over averaged measurement was generated and the position errors were then calculated based on the correlation curves for different placements of diodes (as mentioned in Section 2.C). Another LUT recording the placement of each diode corresponding to its minimum position error was saved and would also be applied for all future measurements. Though the LUT might not represent the real position of diodes inside the ArcCHECK phantom, this step still provided a fast and easy way to minimize the inaccuracy.
Step 3: Since the accuracy of dose consistency and linearity for the diodes used in ArcCHECK were within 1% and 0.5%, and the output consistency of CyberKnife on different day was within 1%, we add 1% margin to the calculated map of relative percentage difference (*RPD*). Based on the three maps: *RPD *− 1%, *RPD,* and *RPD *+* *1%, the minimum position errors of selected diodes would be chosen to present the final results.

Once the two LUTs were generated, the analysis of measurement could be streamlined. MATLAB programs were developed to process these measured dose files (*.txt files*) by ArcCHECK. There was no special tool box or advanced programming skills involved in this study. Analysis results could be obtained in seconds after a 20‐min treatment delivery.

Consecutive measurements were conducted in 6 months to test the performance of our proposed method. Since the routine AQA and E2E tests gave good results, we had no chance to detect the beam path inaccuracy in real situation. Therefore, we introduced shifts of alignment center ranging from 0.3 to 2.0 mm by pulling the Styrofoam insert out in SI direction carefully. To minimize the additional uncertainty from the TLS, we made sure the only change of robot correction after pulling out the insert was the translational shift in SI direction.

### 2.E Verification of correlation curves

Measurements were also conducted to verify the correlation curves of the diodes. During the delivery of the QA plan, we manually stopped the delivery at two selected beam nodes where the response of the diodes was relatively large. Then the position shifts of robot mastering in AP, LR, and SI directions were introduced using teach pendent. The same MUs were delivered at each shift and the measured signals from the corresponding diodes were recorded.

## 3 RESULTS

### 3.A Density override

**Figure 4:**
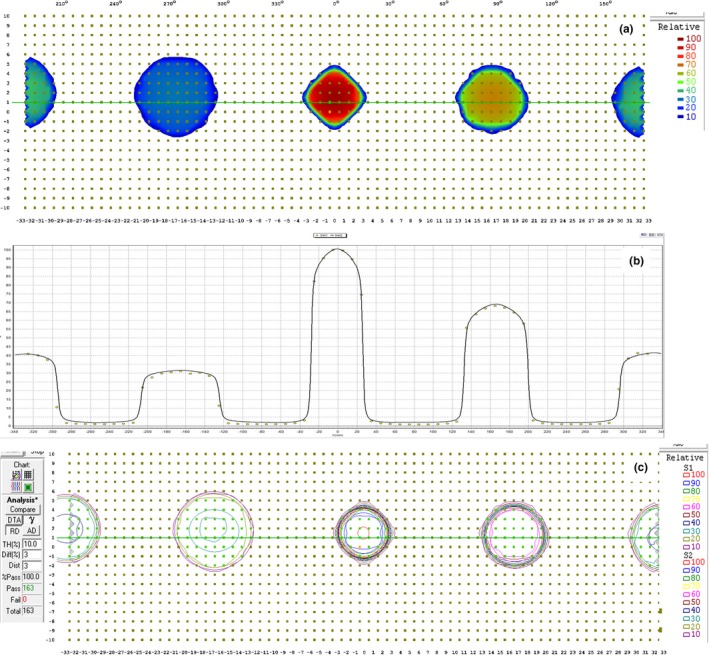
Relative comparison between the ArcCHECK measurement and data calculated from the MultiPlan for two‐field plan: (a) ArcCHECK measured result, (b) Comparison between ArcCHECK measured and MultiPlan calculated profiles, and (c) two‐dimensional dose distributions.

Figure 4 shows the comparison between the measurement and data calculated by the MultiPlan. In order to eliminate the uncertainty from dose calibration of the ArcCHECK, relative comparison was used to evaluate the value of density override. The ratios of entrance to exit dose for both beam paths agrees very well with the calculated values, with a deviation of less than 0.2%, which indicates the assigned RED values of 1.138 for PMMA and 0.19 for Styrofoam are adequate.

### 3.B Verification of correlation curves

**Figure 5:**
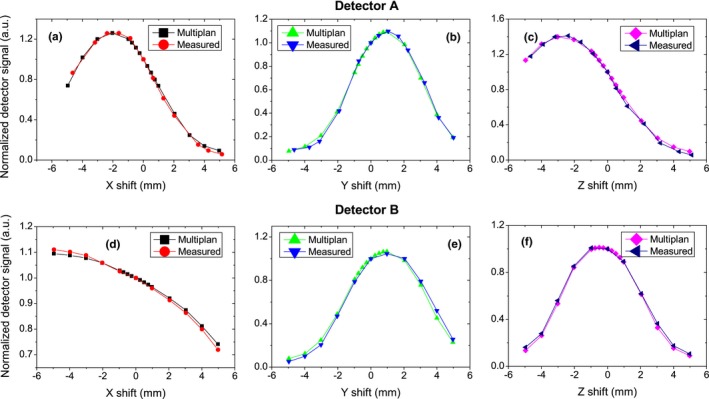
Measured correlation curves in (a) and (d) left–right, (b) and (e) superioinferior, and (c) and (f) anterioposterior directions and their comparisons with those calculated from MultiPlan for two randomly selected diodes.

As illustrated in Fig. 5, the measured correlation curves agree well with the calculated curves, especially within the range of ±1 mm. Though it was not shown in this manuscript, the maximum percent difference between the calculated beam profile by MultiPlan and measured beam profile for 5 mm cone at 15 and 50 mm depth was <0.3%, which verified a very small uncertainty coming from the TPS modeling.

### 3.C Reproducibility

As mentioned in step 2 of Section 2.D, the calculated position errors actually presented the reproducibility of robot mastering. Table 1 shows the root mean square (RMS) and the maximum of those calculated position errors based on 1‐SD and 2‐SD. Machine verification test of beam path accuracy, which is actually the quantitative evaluation of the third level test as discussed in the Section 1 and was conducted just after a full path calibration during machine installation for fixed collimator, is also listed for comparison. Calculation based on 1‐SD gives almost the same maximum deviation as the machine result while its RMS is smaller, which can be explained by the underestimation because of the selection of minimum values during calculation.

**Table 1:** Root mean square (RMS) and maximum of position errors for reproducibility test

	RMS (mm)	Max (mm)
Machine	0.077	0.268
1‐SD	0.054	0.263
2‐SD	0.098	0.382

### 3.D Constancy of beam path accuracy

**Figure 6:**
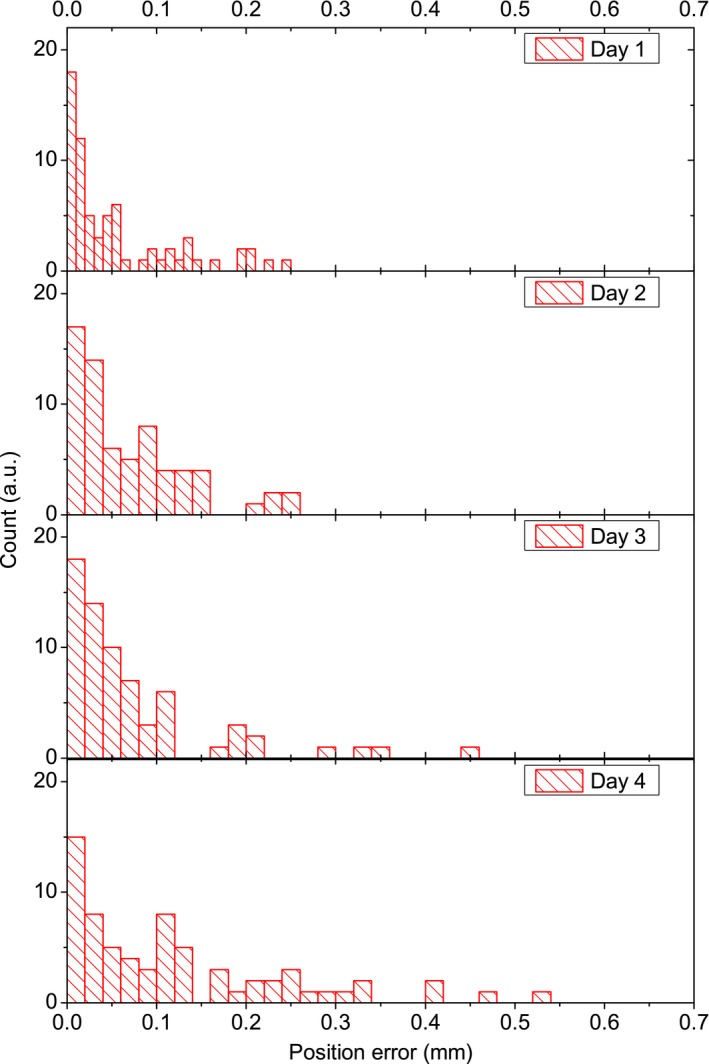
Representative histograms of measured position errors for constancy test.

More than 20 measurements were performed within 6 months and some representative histograms of measured position errors are shown in Fig. 6. The maximum position error for all measurements was within 1 mm. The mean of position errors was 0.091 mm with a SD of 0.035 mm, while the RMS was calculated to be 0.127 mm with a SD of 0.034 mm. Machine verification test for the fixed collimator also showed a comparable result with the RMS of 0.211 mm and the maximum error of 0.492 mm. Figure 6 also seems to show a trend of slowly increasing position error as days increase. This is consistent with the findings of the service engineers during preventive maintenance inspections (PMI) on integrity of beam path calibration of the CyberKnife, and the AQA test results from our center that showed a slight drift from a mean of 0.344 mm with SD of 0.173 mm in the first month after commissioning, to a mean of 0.466 mm with SD of 0.068 mm in the month before the writing of this manuscript. However, at this stage, we do not have enough data (e.g., frequently‐performed machine verification tests) to conclude such finding and further study will be necessary.

### 3.E Capability of detecting position error

**Figure 7:**
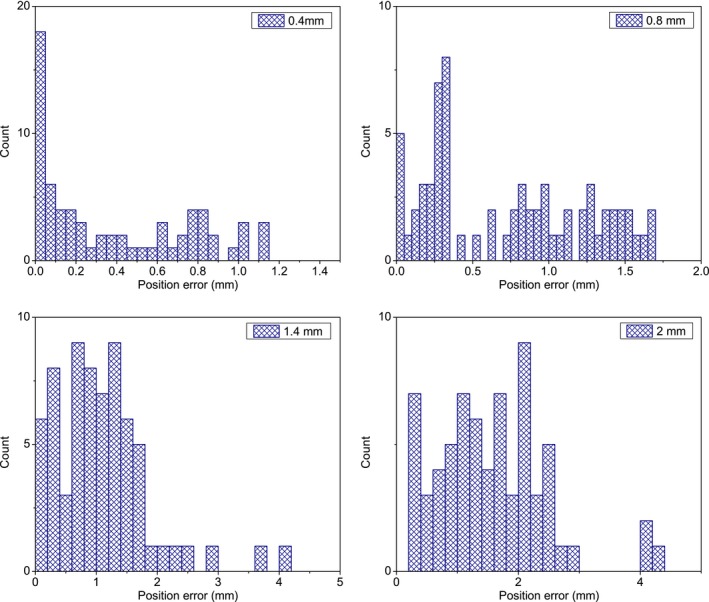
Histograms of measured position errors for different introduced shifts of alignment center.

Figure 7 shows the histograms of measured position errors for different introduced shifts respectively. More than 80% of calculated position errors were in SI and AP direction, which could be explained by many laterally orientated treatment beams as indicated in Fig. 2. For the shift of 2 mm, because some selected diodes in our analysis are more sensitive to the dose variation compared with the laser‐based method, the maximum detected position error using our method (for the worst case) is as large as 4.215 mm. The minimum detectable mean and RMS of position errors (at 95% confidence level) calculated based on the results shown in Section 3.D were 0.173 and 0.239 mm respectively. According to Table 2, it is possible for our method to detect a systematic position error of less than 0.3 mm. Because the number of selected beam is less than the total number of beams, our method may be more sensitive to the position error. Therefore, we think the tolerance of 0.5 mm for RMS is still applicable.

**Table 2:** Mean, root mean square (RMS), and maximum of measured position errors for different introduced shifts of alignment center

Introduced shift (mm)	Mean (mm)	RMS (mm)	Max (mm)
0.3	0.282	0.391	0.814
0.4	0.391	0.538	1.142
0.8	0.733	0.904	1.703
1.4	1.110	1.353	4.091
2.0	1.563	1.721	4.215

## 4 DISCUSSION

**Figure 8:**
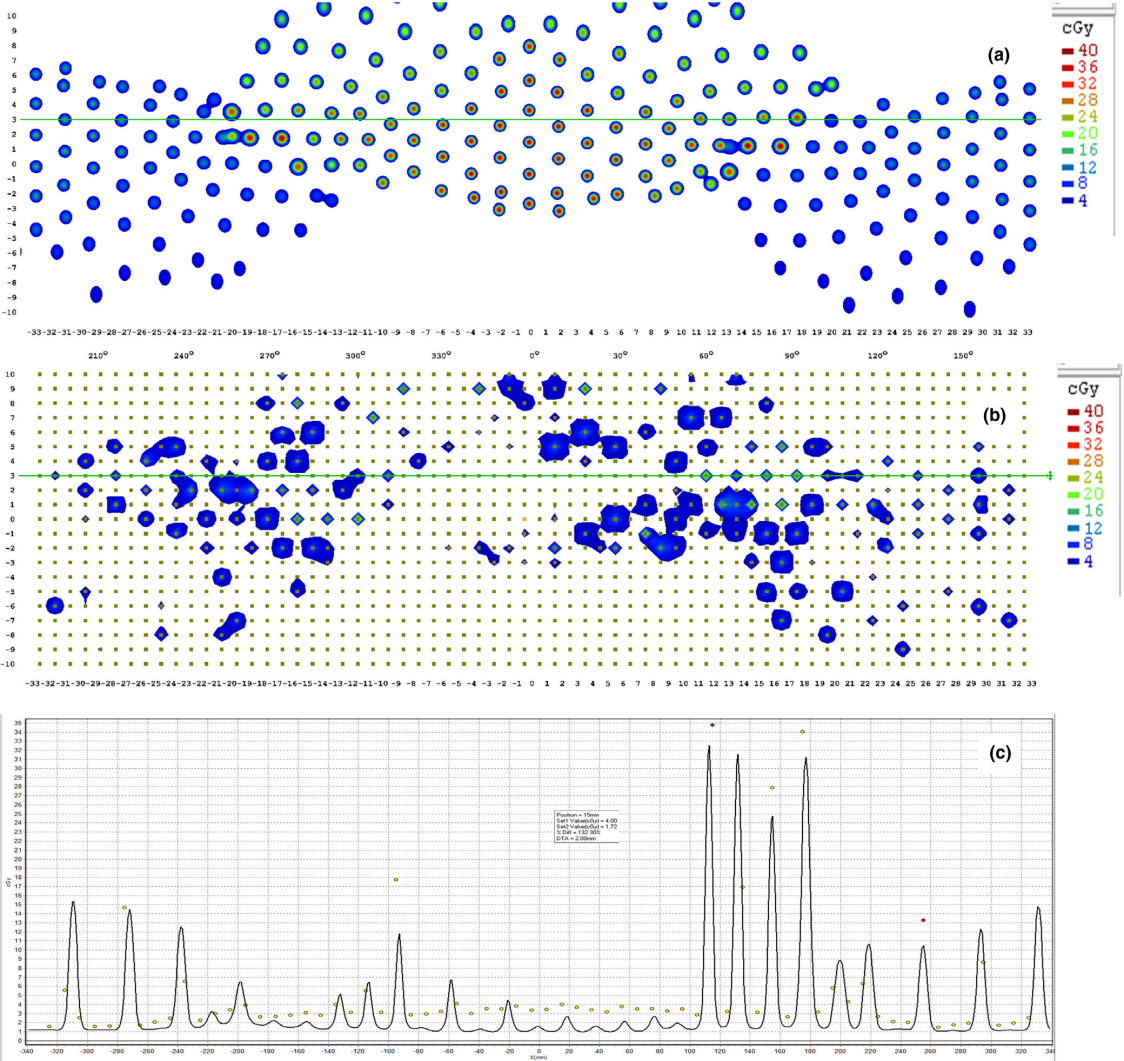
(a) Calculated dose distribution by MultiPlan; (b) measured result by ArcCHECK; (c) profile comparison.

**Figure 9:**
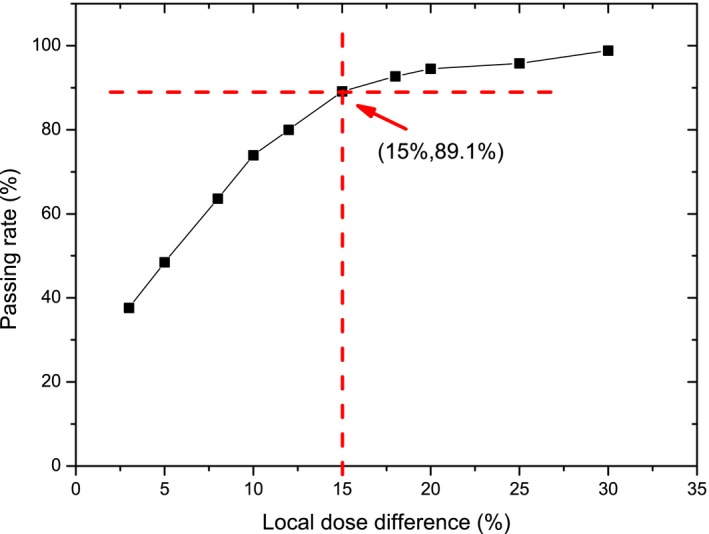
Plot of dose to agreement passing rate as a function of local dose difference for data measured on two different days.

As shown in Fig. 8, it is inappropriate to directly compare the measurement with the dose distribution calculated by MultiPlan. Not only some beams were partially or entirely missed because of the 10 mm detector spacing of the ArcCHECK, but the angular and dose rate dependence of the ArcCHECK made the direct comparison difficult. Dose to agreement (DTA) analysis was also conducted for measurements taken on different days. As indicated in Fig. 9, the local dose difference had to be increased to ~15% to achieve a passing rate higher than 90%, which means 10% of the selected detectors still have dose variations larger than 15% during the consistency check. Compared with conventional DTA analysis that only gives a single value of passing rate, our proposed method is able to calculate the position shift for each single beam and is more suitable for the evaluation of path accuracy.

As described in step 3 of Section 2.D, 1% was chosen as the additional margin for taking the diode performance and output consistency into consideration. In fact, the total uncertainty can be larger than 1% after taking the uncertainty of TLS into account. It is reasonable and conservative to select this value since we have chosen the minimum value as the final position error. Some studies mentioned the dose‐per‐pulse dependence of ArcCHECK diodes,^21^ which could be as large as 3% when SDD changes from 100 to 80 cm. However, this effect will not affect our results because our measurements were compared with the baseline instead of TPS calculation. The proposed test is not designed for determining the absolute position of each beam, but a constancy test for measuring the deviations of beam paths from the original state which was taken as the baseline after the robot mastering was calibrated and verified. Compared with the BB test, our method is able to provide quantitative analysis result for individual beam.

The rationale behind step 1 and 3 described in Section 2.D is that the CyberKnife is proven to be very stable by many centers.^8^, ^22^, ^23^, ^24^, ^25^ The possibility of a sudden and large drift of the robot mastering is very low during routine operation and treatment. In addition, though the position errors in three directions of AP, SI and LR were calculated for convenience, the final result may still underestimate or overestimate the so‐called “true” drift due to the complication of geometrical relation between beams and the ArcCHECK in 3D space. Therefore, it should be emphasized that the minimum position error calculated based on the proposed method is a parameter for evaluating the constancy of beam path accuracy, which does not represent a global minimum.

In this study, we have verified the feasibility of the proposed QA method for fixed collimator only. Similarly, this method may apply to both Iris and Multileaf Collimator (MLC) collimators as well. However, since the minimum applicable cone size for Iris in an isocentric plan is 7.5 mm and the claimed leaf position accuracy for treatments using the MLC collimator is better than 0.95 at 800 mm SAD, the resulted sensitivity and accuracy may not be as good as that for fixed collimator with 5 mm cone, which needs to be investigated in the future. BB test has no recommendation or requirement of the collimator used for creating a QA plan. We believe, as a QA procedure for checking constancy of beam paths, our test has served the purpose at current stage. We should emphasize that the proposed QA method was not developed to substitute the well‐established E2E tests but focus on the quantitative evaluation of beam path accuracy. If the result of RMS is out of the tolerance of 0.5 mm, E2E tests still need to be performed to make sure that the overall targeting accuracy of the system is within the tolerance for clinical treatment. We should also make it clear that our results included the uncertainty of TLS. Though QA checks of TLS and couch movement correspondence^19^ have been performed periodically using calibrated ruler and inclinometer, the precision of those tools for evaluating the translational and rotational corrections was limited to 0.2 mm and 0.1°. After taking the geometry of ArcCHECK into consideration, the inherent uncertainty of our method may be as large as 0.26 mm that was comparable to the minimum detectable error calculated in Section 3.E. Therefore, unless the accuracy of TLS has been confirmed, any bad result obtained in our proposed test may indicate either a drift of TLS accuracy or a drift of beam path that needs a recalibration in the next PMI.

Though the isocentric plan used a full body path with a total of 116 beams, our study only included 68 diodes/beams. It was expected because some beams would be totally missed due to the limited special resolution of ArcCHECK. In addition, we also had to filter out those diodes irradiated by opposing or closly opposing beams, which would cancel out or exaggerate the calculated error. It is obviously that our current method is not able to evaluate the full beam path, and a true 3D dosimeter, for example, gel dosimetry system, should be more suitable for studying the targeting accuracy. However, this study was a proof of concept about how to utilize the unique 3D structure of a widely‐used dosimetry QA tool for machine QA of CyberKnife.

## 5 CONCLUSION

This study has proposed a novel and convenient QA method for evaluating the constancy of beam path accuracy of CyberKnife, which also makes the assessment of position accuracy for single beam possible. Compared with the recommended method by the manufacturer, our proposed method is efficient and suitable for routine QA with high sensitivity.

## CONFLICT OF INTEREST

The authors declare no conflict of interest.
